# Supplementary material for: The cytokine GDF15 signals through a population of brainstem cholecystokinin neurons to mediate anorectic signalling
Source: eLife. 2020 Jul 29;9:e55164. doi: 10.7554/eLife.55164 (PMC7410488; doi:10.7554/eLife.55164)
Supplement: Supplementary file 1. — The number of cells per section single-, double- or triple-labelled on sections through the AP and NTS. Values are stated as are mean ± SEM (n = number of animals). Percentage co-expression is written in the text. Methods involved either immunohistochemistry (top) or in situ hybridisation histology (bottom). [file elife-55164-supp1.docx]

| **Immunohistochemistry** | **Cells per section (mean ± SEM)** | | **n (mice)** | **Corresponding Figure** |
| --- | --- | --- | --- | --- |
|  | **AP** | **NTS** |  |  |
| GFRAL | 22 ± 1 | 21 ± 1 | 4 | Fig 1B  + Suppl Fig 1B |
| *Cck*^Cre::eYFP^ | 67 ± 5 | 175 ± 10 |  |  |
| TH | 27 ± 1 | 52 ± 3 |  |  |
| GFRAL + *Cck*^Cre::eYFP^ | 13 ± 2 | 7 ± 1 |  |  |
| GFRAL + TH | 6 ± 1 | 10 ± 0 |  |  |
| *Cck*^Cre::eYFP^ + TH | 8 ± 2 | 11 ± 1 |  |  |
| GFRAL+ *Cck*^Cre::eYFP^ + TH | 3 ± 0 | 3 ± 0 |  |  |
| GFRAL | 19 ± 2 | 18 ± 1 | 4 | Fig 1B |
| *Prlh*^Cre::eYFP^ | 1 ± 0 | 25 ± 1 |  |  |
| GFRAL + *Prlh*^Cre::eYFP^ | 0 ± 0 | 0 ± 0 |  |  |
| GFRAL | 20 ± 1 | 20 ± 1 | 4 | Fig 1B |
| *Gcg*^Cre::eYFP^ | 0 ± 0 | 23 ± 2 |  |  |
| GFRAL + *Gcg*^Cre::eYFP^ | 0 ± 0 | 0 ± 0 |  |  |
| GFRAL | 19 ± 2 | 20 ± 1 | 5 | Suppl Fig 1C |
| *Slc17a6*^Cre::eYFP^ | N/A | N/A |  |  |
| GFRAL + *Slc17a6*^Cre::eYFP^ | 10 ± 2 | 4 ± 1 |  |  |
| *Cck*^Cre::eYFP^ | N/A | 119 ± 8 | 2 | Suppl Fig 1D |
| GLP-1 | N/A | 13 ± 1 |  |  |
| *Cck*^Cre::eYFP^ + GLP-1 | N/A | 2 ± 0 |  |  |
| ***In situ* hybridisation** | **AP** | **NTS** | **n (mice)** | **Corresponding Figure** |
| *Gfral* | 25 ± 3 | 19 ± 2 | 3 | Suppl Fig 1A |
| *Cck* | 37 ± 5 | 81 ± 7 |  |  |
| *Gfral* + *Cck* | 17 ± 2 | 7 ± 2 |  |  |

LUCKMAN eLife - Supplementary File 1
